# Beyond the Visual Spectrum: From RGB-Based Learning to Hyperspectral Intelligence for Plant Disease Detection—Challenges and Opportunities

**DOI:** 10.3390/s26123834

**Published:** 2026-06-16

**Authors:** Muhammad Hanif Tunio, Shaowen Li, Awais Ahmed, Liu Lei, Changyong Liang

**Affiliations:** 1 School of Big Data and Artificial Intelligence, Anhui Xinhua University, No. 555, West Wangjiang Road, Hefei 230088, China; hanif@axhu.edu.cn (M.H.T.); liulei01@axhu.edu.cn (L.L.); 2 School of Artificial Intelligence, Anhui Agricultural University, No. 130, West Changjiang Road, Hefei 230036, China; 3 School of Computer Science, China West Normal University, Nanchong 637002, China; ahmedawais@cwnu.edu.cn; 4 School of Management, Hefei University of Technology, No. 193, Tunxi Road, Hefei 230009, China; cyliang@hfut.edu.cn

**Keywords:** plantdisease, RGB, hyperspectral imaging, deep learning, controlled and field environments, multimodal fusion

## Abstract

Plant diseases result in the estimated loss of 20–40% of the world’s crop production annually, amounting to more than $220 billion in economic losses and threatening food security for a rapidly expanding world population. While the conventional methods for detecting plant diseases rely on visual inspection of the symptoms, they are resource-consuming. For effective plant disease detection at a pre-mature stage, hyperspectral imaging (HSI) represents a paradigm shift in technology. It can be used to obtain subtle spectral signatures outside the visible spectrum, which enables pre-symptomatic and highly specific plant disease diagnosis. Concurrently, deep learning (DL) has become the prevalent analytical paradigm for decoding the complex and high-dimensional data that HSI produces. This paper covers a comprehensive narrative review of the intersection of these two transformative technologies from 2008 to 2026. We first set out the biological and physical principles by which HSI is uniquely suited to detecting plant–pathogen interactions in the absence of visible symptoms. We then present a detailed taxonomy of deep learning architectures for Vision Imaging and HSI data, ranging from basic 1D and 3D convolutional neural networks (CNNs) to hybrid models with attention mechanisms and, most recently, vision transformers, which have achieved greater robustness to real-world conditions. There is currently a major and consistent “lab-to-field” performance gap. A critical analysis of various studies reveals a persistent and significant performance gap between models that perform well on controlled lab datasets (ranging from 95 to 99%) and field-collected data (typically 70–85%). This paper also addresses the practical gap of environmental variability, image noise, and the domain gap between the controlled environment and the real dataset. Finally, this review concludes by providing strategic research recommendations and a roadmap, highlighting that the future of the field is contingent upon not only architectural innovation but also a holistic approach, with robustness, scalability, affordability, and interpretability as the main focus to bring the proven potential of HSI-DL systems from the lab to the field, ultimately contributing to global food security.

## 1. Introduction

### The Unrelenting Threat of Plant Diseases

Agriculture is a mainstay of national economies and a key component of world food security, especially in developing countries, where it is a significant contributor to GDP and employment. Agricultural systems are a source of vital resources such as food and fiber and are responsible for maintaining nutritional needs as well as economic stability. However, the productivity of these systems is increasingly compromised by plant diseases, which account for an estimated 20–40% of annual crop losses worldwide, with serious financial damages reaching beyond USD 220 billion per year [1,2,3,4]. Table 1 illustrates the impact of plant on different crops in the world. The sense of urgency in this challenge is also increased by the rapid growth of the global population, estimated by the United Nations to reach 8.6 billion by 2030, 9.8 billion by 2050, and 11.2 billion by 2100 [5]. To ensure the needs of this growing population, the Food and Agriculture Organization (FAO) has estimated that agricultural production needs to increase by 70–80% by 2050 [6,7]. These projections highlight the urgent need for innovations to enhance agricultural productivity, resilience, and sustainability.

To realize these objectives, early and precise identification of plant diseases is critical to mitigating excessive pesticide use, minimizing labor costs, and enabling timely intervention [8,9]. The conventional approaches of detecting plant diseases include expert inspection and lab-based molecular diagnostics, which are time-consuming, costly, and not accessible to small farmers, among other factors, depending on the detection methodology used at a specific time and location [10,11,12,13,14]. These shortcomings have influenced the implementation of automated, scalable models based on artificial intelligence (AI), specifically vision-based machine learning (ML) and deep learning (DL) systems, which have shown impressive accuracy in the classification of plant diseases using leaf images [15,16,17]. The latest developments in ML and DL models, such as convolutional neural networks (CNNs), generative adversarial networks (GANs), attention head mechanisms, and vision transformers (ViTs), have transformed the field of plant pathology as they can detect diseases quickly, non-invasively, and on a large scale using large quantities of data [18,19,20]. These approaches are mainly based on annotated image datasets like PlantVillage, PlantDoc, and AgroDataset to generate techniques that have the capability to recognize disease symptoms based on leaf texture, shape, and color patterns.

However, the vast majority of current models are trained on controlled, laboratory-quality images and perform poorly in field scenarios with occlusion, changing illumination, and mixed infections, reducing accuracy from 95–99% on laboratory dataset images to 70–85% in complex field settings [21,22,23]. This discontinuity shows one of the fundamental weaknesses: traditional RGB-based techniques are applied to capture only the effects of surface-level visual symptoms, which can only be expressed once the plants have already been damaged to a significant extent, physiologically speaking.

HSI is a transformative alternative to the visible spectrum [24,25]. HSI enables the detection of subtle biochemical and physiological changes in the tissues of the plant long before they are visibly detected due to the capability to continuously measure reflectance over hundreds of spectral bands across the visible, near-infrared (NIR), and shortwave infrared (SWIR) spectral bands [26,27,28]. This pre-symptomatic diagnosis, which is often possible before symptoms of the disease are evident, has colossal potential in the early intervention and accurate treatment of diseases themselves, which contributes to sustainable agriculture by reducing unnecessary use of pesticides and reducing losses in crops [27,29]. Hyperspectral imaging and deep learning are promising but highly challenging when it comes to integrating the two methods. The high dimensionality, spatial–spectral correlation, and high redundancy define hyperspectral data cubes, and special architectures, including 3D CNNs and hybrid spectral–spatial models, are needed to analyze them [30,31,32]. Moreover, the high price of hyperspectral cameras and the processing power needed to process large spectral datasets also complicate the deployment of hyperspectral cameras in the field even more [33,34]. Additionally, the scarcity of publicly available annotated hyperspectral datasets limits model generalization and cross-crop validation [35]. Current solutions often fail to address the practical realities of on-site deployment, particularly in remote or resource-constrained agricultural settings.

**Table 1:** Global impact of major plant diseases across different crops.

Crop	Disease	Region	Impact	Year	Reference
Wheat	Wheat Blast	Bangladesh	8205 tons wheat loss (~USD 2.1 million)	2016	[36,37]
Maize	Maize Lethal Necrosis (MLN)	Kenya	~60,000 ha affected (~USD 50 million loss)	2013–2014	[38,39]
Coffee	Coffee Leaf Rust (CLR)	Ethiopia, Uganda	Up to 49.5% yield loss; ~USD 1 billion impact; ~250,000 jobs lost	2012–2013	[40,41]
Citrus	Citrus Greening (HLB)	North America (USA)	~USD 3.6 billion annual losses	2005–present	[42]
Olive	*Xylella fastidiosa*	Europe (Italy, Spain)	>USD 1 billion economic damage	2013–2019	[43,44]
Potato	Late Blight	Global	USD 3–10 billion annual losses	Ongoing	[45]
Tomato	Early Blight	Europe, Africa, India	Up to 80% yield loss	Various	[46]

The convergence of deep learning with edge computing platforms, mobile devices, and emerging multimodal AI frameworks offers pathways to democratize access to advanced diagnostics. It is worth mentioning that although the practice of mobile-enabled plant disease detection systems has been proven possible by the example of applications like Plantix and AgriApp, all of them turn out to be inconsistent in their performance in real-field settings, and their performance based on RGB images also limits the ability to detect plant disease before it progresses to the symptoms stage of the disease process [47,48]. In addition to vision-based systems, with the introduction of Large Language Models (LLMs) like GPT-4 and vision–language models, human–AI interaction in agriculture has taken on a new dimension of interaction with AI [49]. These models may be used to autotag hyperspectral data, produce actionable disease prevention advice based on spectral features, and analyze a complex stream of multimodal data. Although the integration of LLMs into the hyperspectral agricultural pipeline is still in its nascent stages, the area has the potential to impact knowledge-based diagnostics, translation into farmer queries, and real-time advisory systems.

**Figure 1:**
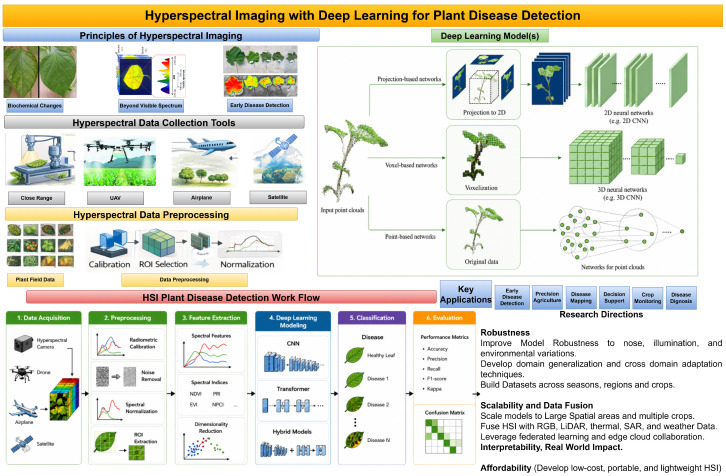
Overview of hyperspectral imaging and deep learning-based approaches for plant disease detection, illustrating principles of spectral sensing, data acquisition platforms, preprocessing pipeline, model development, key applications, field challenges, and future research directions.

Table 2 highlights the limitations of existing review studies, which primarily focus on either RGB or hyperspectral approaches without addressing the lab-to-field performance gap or providing a unified analytical framework. In contrast, this work introduces a comprehensive (narrative) review, integrating sensing modalities, data challenges, and model generalization into a structured perspective. Furthermore, this review also analyzes the opportunities as well as the ongoing challenges of the visual spectrum. We comprehensively studied the existing spectral–spatial deep learning architectures, such as 3D CNNs, 2D/3D hybrid models, and transformer-based models, created over the years, and discussed the existing vision and hyperspectral datasets, gaps in the methodology, model interpretability, domain adaptation, and lightweight deployment. Unlike the current literature, which mainly addresses RGB-based solutions, this paper demonstrates a dedicated overview of vision and hyperspectral deep learning, incorporates insights of emerging multimodal and LLM-based frameworks, and suggests a research roadmap for the construction of accessible, scalable, and sustainable plant disease detection systems for practical use in agriculture. Figure 1 displays the overall systematic description of this review to demonstrate the pipeline that was followed during this research.

## 2. Approach of Comprehensive (Narrative) Review

This section outlines the approach used to conduct this review, which provides a clear, transparent description of the existing literature search strategy and the selection process for identifying and evaluating relevant studies. The primary objective is to conduct a comprehensive comparative analysis of existing and recent research, critically assess their findings, and identify key limitations and gaps in the current body of knowledge in relation to research questions.

### 2.1. Comprehensive Literature Search and Selection Process

This comprehensive review was conducted in three interconnected stages: input, process, and output.

During the first phase, a preliminary scoping review of the literature and discussion with domain experts were used to develop research questions. The given procedure allowed identification of the main research gaps and limitations of the existing literature, which led to the creation of specific research questions, the purpose of which was to analyze the area, assess the current methods, and determine possible future directions of research. To fulfill these goals, a logical and strict literature search was implemented in large academic databases, such as Google Scholar, Scopus, and Web of Science. Only peer-reviewed articles published in 2008–2026 were searched. To ensure maximum coverage of the relevant studies, a set of carefully selected keywords was used that relates to plant disease detection with the use of deep learning. The search strategy was tailored to respond to the research questions and identify old and recent contributions in the area. In addition, the chosen articles were also cross-authenticated with their source of publication to ascertain accuracy, authenticity, and completeness.

After the initial screening of studies, a two-stage screening was performed. Titles and abstracts were initially screened to eliminate irrelevant articles, and then full-text screening was conducted to consider studies that satisfied the pre-specified inclusion criteria. The eligibility rate, including the number of records screened, evaluated, and incorporated into the final review, was determined. Data extraction was performed in order to maintain uniformity and precision by use of a standardized form that would extract important data of each study, such as study design, character of the data, source of data, and key findings. Moreover, quality evaluation of the incorporated studies was conducted to assess the structured rigor and risk of bias. Two reviewers conducted this process independently, and any differences were discussed and agreed upon. To guide this comprehensive review, the following research questions (RQs) were developed:

RQ1. How can the lab-to-field generalization gap of RGB-based deep learning models be minimized under varying illumination, occlusion, and background complexity?
RQ2. To what extent can hyperspectral imaging enable robust pre-symptomatic disease detection across diverse crop species and disease stages?
RQ3. How can multimodal fusion of RGB, hyperspectral, thermal, UAV, and environmental sensing be optimized for reliable large-scale field deployment?
RQ4. What benchmark design principles are required to develop standardized public RGB–HSI datasets with temporal disease progression and geographically diverse field conditions?
RQ5. How can lightweight transformer and edge AI architectures maintain high diagnostic accuracy under real-time smartphone, UAV, and IoT-assisted deployment constraints?
RQ6. What role will emerging vision–language and foundation models play in improving explainability, transferability, and cross-crop disease diagnosis?

### 2.2. Publication Trends and Bibliometric Analysis of Plant Disease Detection

The comprehensive (narrative) review of the articles published between 2008 and 2026 shows that there is a definite shift towards more innovative deep learning and hyperspectral imaging models and mechanisms of machine learning. The initial research was mainly based on hand-crafted feature-based machine learning methods using RGB images, which were usually susceptible to environmental variations and limited to model generalization. As deep learning has rapidly evolved, especially in convolutional neural networks and transformer-based models, the volume of publications has increased significantly, with a focus on achieving better results in automated learning of spatial features of large datasets. Trends over time show that after 2017, the increase in research output is steep due to the presence of benchmark datasets and the availability of more computational resources, while contributions by country contain a lot of activity in those regions that invest in precision agriculture and those that rely on AI-based solutions.

Furthermore, the progress does not suffice to ensure that deep learning with RGB techniques is no longer limited to the visible spectrum, which restricts its use in detecting disease in its early stages. In more recent developments, the combination of hyperspectral imaging and deep learning has become a prospective research area, which allows recording and analyzing spectral–spatial signatures in more detail than those visible to human eyes. This trend can be observed in the growing number of recent papers dedicated to the use of hyperspectral data and multimodal learning [59]. Hyperspectral deep learning methods can improve diagnostic strength and enable earlier detection of subtle biochemical and physiological alterations before the development of visible symptoms of the plant diseases, making them more robust and capable of earlier diagnosis [27,60].

#### 2.2.1. Search Mechanism

A comprehensive bibliometric analysis was conducted to examine and evaluate the development of research on the detection of plant diseases. The relevant publications were obtained from large scientific databases, such as Scopus, Web of Science, and Google Scholar, during the period 2008–2026. A combination of keywords using machine learning, deep learning, RGB imaging, and hyperspectral imaging was used in detecting plant diseases based on research studies in the search strategy of this review. In particular, some of the search queries were “plant disease detection”, “machine learning”, “deep learning”, “convolutional neural networks”, “vision transformers”, “RGB imaging”, “hyperspectral imaging”, and “precision agriculture”. The search was further refined with the help of the Boolean operators (AND, OR) in order to have complete coverage.

(1) $$\mathrm{Growth}\;(\%)=\;\frac{{\mathrm{Publications}}_{\mathrm{current}}-{\mathrm{Publications}}_{\mathrm{previous}}}{{\mathrm{Publications}}_{\mathrm{previous}}}\times 100$$

(2) $$\mathrm{Adjusted}\;{\mathrm{Publications}}_{2026}=\frac{1530}{3}\times 12=6120$$

**Figure 2:**
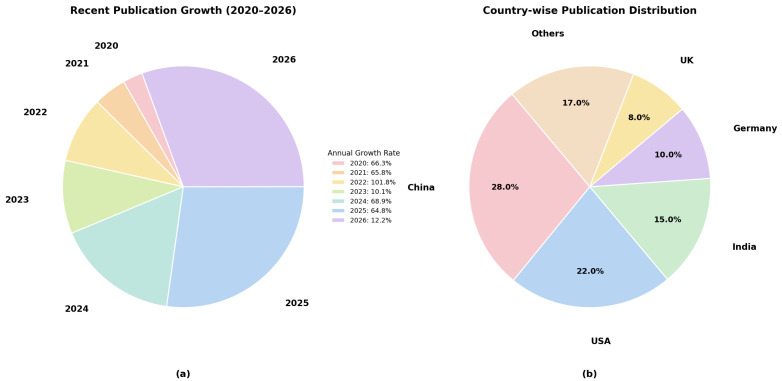
(**a**) Pie chart showing the partial temporal distribution of research publications on plant leaf disease detection from 2020 to 2026; the value for 2026 represents an annualized projection derived from the first-quarter publication count using Equation (2). (**b**) Country-wise distribution of publications, emphasizing contributions from China, the USA, India, Germany, the UK, and other regions in this research domain.

To analyze temporal research trends, the number of publications was collected every year and divided by temporal progression and number of publications, as presented in Table 3; country-wise contributions are shown in Table 4 and visually represented in Figure 2, which represents that China is the major contributor in the field of plant disease detection, the values in the table were computed by first author affiliation and the percentage was derived by the number of papers in a specific country divided by the total number of papers published in the field. Furthermore, an analysis of keyword co-occurrence was conducted to identify prevailing research topics and emerging issues in the field. The polished dataset helped to identify key research areas, which included vision-based disease detection, spectral analysis, and multimodal learning strategies. This structured way of providing a strong and replicable evaluation of the research space shows the fast shift from traditional machine learning strategies to deep learning and solutions based on hyperspectral imaging.

#### 2.2.2. Search Keywords Analysis

Table 3 summarizes the temporal change in publications in the area of plant disease detection between 2008 and 2026. To get a fair comparison of the results against the past full-year results, the partial publication number, 1530 as the first quarter of 2026, was annualized with Equation (2). The values were projected on the basis of the first three months, which were multiplied by the ratio of a full year, which was projected to be 6120. Such normalization allowed a consistent year-by-year growth analysis through the entire historical dataset, where the growth percentage of publications was determined through Equation (1). This literature was the foundation of further analysis by means of the key words co-occurrence, which revealed the thematic evolution of the sphere. Finally, the total amount of keyword co-occurrences identified in the publications collected was 16,750 (excluding projected values for 2026), taking into account that at least five keywords occurred together in the titles, abstracts, or keywords list. The dataset was narrowed down to 200+ co-occurrences, upon which a more comprehensive analysis was possible, after refining the dataset with the help of a thesaurus to eliminate the occurrence of redundancy and the need to unify terminology.

## 3. Conventional Machine Learning and Deep Learning Approaches

Research on machine learning for plant disease identification gained momentum approximately two decades ago, as agricultural applications began attracting widespread attention [61]. Early efforts relied on traditional ML algorithms: support vector machines (SVMs) for tomato diseases, random forests for tomato disease detection [61], meta-knowledge transfer [62], and K-nearest neighbors (KNNs) for soybean diseases [63]. These techniques extended beyond identification to severity estimation; for instance, SVMs, KNNs, and Naïve Bayes were used to detect and forecast tomato powdery mildew [64].

However, the conventional ML approaches have various limitations since they require feature extraction to be done manually, which is a rather time-consuming process, and these methods perform well under controlled and seen patterns of data [65,66]. This constraint led to a paradigm shift to deep learning that automatically extracts hierarchical features out of datasets.

The turning point was with the 2012 ILSVRC ImageNet competition [67], which indicated the transformational capabilities of deep convolutional neural networks (CNNs). CNNs have played the role of the workhorse in plant disease detection, performing in classification, object detection, and semantic segmentation [68]. Hybrid schemes that combine segmentation and classification have even enhanced the accuracy of detection [69].

Large datasets with high quality are critical in the success of deep learning [70]. In 2015, the field of plant disease detection was catalyzed with the release of PlantVillage [71], which is a benchmark to identify diseases, estimate their severity, and develop detection and control systems. Later on, more datasets such as Digipathos, PlantDoc, Northern Leaf Blight (NLB), RoCoLe (coffee), rice disease, and cassava disease became publicly accessible, allowing broader model training and validation [72,73,74]. Nowadays, deep learning is characterized by specific benefits compared to traditional methods: it is able to extract features automatically, detect various diseases simultaneously, draw precise severity estimates, and it can be deployed cost-effectively and on a large scale [75]. However, these benefits are limited when they are used in traditional RGB imagery, which can identify the disease of plants at their maturity stage.

### Limitations of Traditional and RGB-Based Detection Paradigms

In the past, farmers or trained pathologists have relied nearly exclusively on visual inspection to detect the presence of plant diseases. This method is well known, inherently tedious, time-consuming, and subjective, with certain limitations that make it impractical for large and commercial agriculture [21,22]. More importantly, the process of visual examination is dependent on the manifestation of observable symptoms like lesions, chlorosis, and wilting, which, in most cases, can be seen in the middle and terminal phases of the infection [60]. When the symptoms can be seen, the pathogen has already extended widely in the plant and to other crops, making intervention much less effective [60,76].

**Figure 3:**
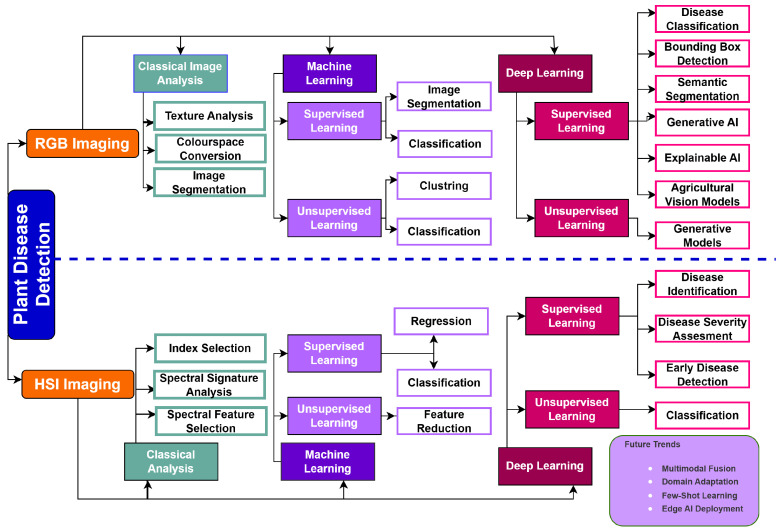
Taxonomy of plant disease detection methods using RGB and hyperspectral imaging, including machine learning and deep learning approaches, illustrating the progression from conventional frameworks to advanced data-driven paradigms, which can be summarized as a lab-to-field performance gap, the figure was inspired by [22].

Computer vision and digital imaging have gained considerable attention from researchers, enabling significant advancements. RGB imaging, using traditional smartphones or digital cameras, made it possible to develop automated systems for classifying diseases, especially with the emergence of deep learning. The best results with convolutional neural networks (CNNs) were recorded in the seminal work by [15], which showed high accuracy (over 99%) on the PlantVillage dataset, a large dataset comprising laboratory-captured images of uniformly colored leaves using convolutional neural networks (CNNs). The breakthrough was followed by a wave of research on RGB-based detection, where other researchers also achieved similarly high accuracies with architectures like VGG, ResNet, Inception, and EfficientNet [61,63,64]. But the intersection and inherent limitation of machine learning (ML)- and deep learning (DL)-based RGB imaging is that they are spectrally confined. Figure 3 represents the detailed taxonomy of plant disease detection methods for RGB and Hyperspectral imaging.

**Figure 4:**
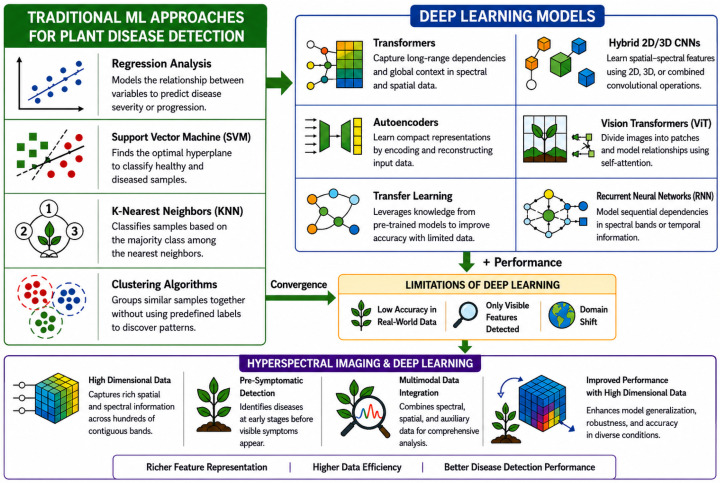
Convergence of ML to DL (conventional images) for plant disease detection and need for hyperspectral imaging.

As Figure 4 shows, RGB sensors only receive three broad bands of the visible spectrum (approximately 400–700 nm), which give data mainly regarding the pigments of plants and their overall morphological transformation. They are unaware of small pre-symptomatic physiological changes that take place in pathogenesis, alterations in the water content of leaves, cellular structure, and biochemical composition, which appear in the near-infrared (NIR, 700–1300 nm) and short-wave infrared (SWIR, 1300–1500 nm) bands. This spectral blindness is why the performance of the models trained on RGB and tested in field conditions is dramatically worse than when operating in a laboratory environment; some research cases are shown in Table 5, where PlantVillage Acc. refers to the classification accuracy of each of the models on the PlantVillage dataset, which is the controlled environment dataset. while reflecting the respective model performance on the data of the PlantDoc or field environment, corresponding to real-life conditions. Acc Drop is a measure of performance degradation in models when the model is used in the transition from laboratory to real-world settings. This lab-to-field gap, often ranging from 15 to 40% and reaching as high as 50–66% for some models, is a major obstacle to the practical usefulness of RGB-based systems.

The quantified impacts presented in Table 6 are derived by aggregating the accuracy drops reported in Table 5. Specifically, the minimum–maximum ranges and subgroup trends were calculated across different model categories to highlight the severity and variability of the lab-to-field performance gap.

## 4. Fundamental Concepts of Hyperspectral Imaging (HSI) Sensors

Hyperspectral imaging (HSI) is a highly effective and diverse tool of analysis that has been developed in the last several decades and it is applicable in various fields of scientific and engineering activity [84]. The technology was initially created to combine spectroscopy and imaging so that both spatial and spectral data can be obtained at the same time [32]. In this combination, a precise identification and mapping of material composition can be determined using its spectral signature. Hyperspectral imaging has a huge advantage over conventional RGB imaging in that it records information in hundreds of adjacent spectral bands. The spectral resolution is rich and enables more detailed characterization of the spatial distribution of biochemical and structural components in a scene. Although both RGB and hyperspectral imaging methods can be used in a non-destructive mode, HSI is the only technique that allows visualization and separation of the chemical compositions on a far more detailed level. When applied to plant disease identification, HSI can give them superior sensitivity to small physiological and biochemical variations that cannot be seen by other imaging techniques. This section presents the basics of the hyperspectral image sensors, their classification, and the platform that can be used to obtain the hyperspectral data in agriculture [27,85].

### 4.1. Principles of Hyperspectral Imaging Sensors

**Figure 5:**
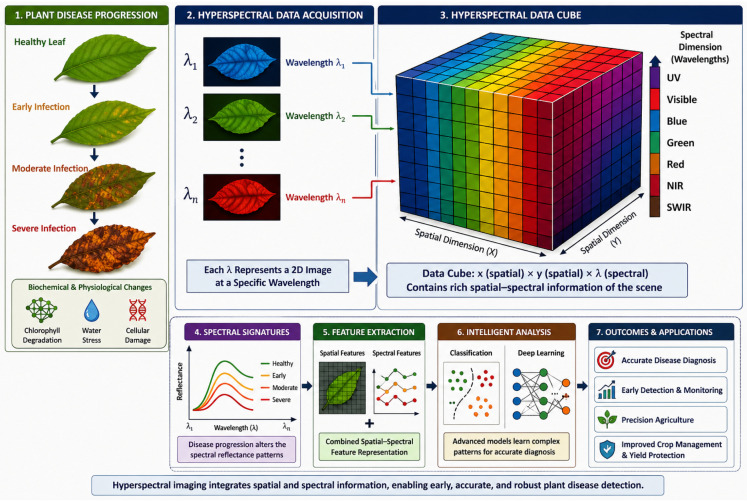
A three-dimensional hyperspectral data cube (hypercube) composed of two spatial dimensions (*x*, *y*) and one spectral dimension (λ), alongside the extracted spectral signature showing reflectance values across different wavelengths for a single pixel.

HSI allows simultaneous acquisition of both spatial and spectral data at a large number of adjacent wavelengths, generally of remote sensing sources. HSI offers the ease of characterizing, in detail, physical characteristics (e.g., size, shape, color, etc.) and chemical composition by combining spectroscopy with imaging. Hyperspectral systems have hundreds of spectral bands compared to multispectral imaging, which only uses a small number of discrete spectral bands, resulting in a high spectral resolution [86]. The operating principle of hyperspectral sensors is analogous to conventional RGB cameras, which measure reflected light in three broad bands. In contrast, hyperspectral sensors acquire reflectance information across hundreds of wavelengths, typically measured in nanometers, resulting in a detailed spectral profile (spectral signature) for each pixel [87]. These spectral signatures are closely associated with the biochemical properties of plant tissues. In plant disease detection, variations in spectral signatures between healthy and diseased plants arise due to changes in pigment composition, cellular structure, and water content. These biochemical and physiological alterations influence leaf color, morphology, and transpiration, which can be effectively captured using HSI [88]. Hyperspectral data are generally modeled as a three-dimensional data cube (hypercube), with two spatial dimensions and a single spectral dimension in the form of a hypercube, as in Figure 5, whereby each pixel of the hypercube has a single spectral signature, neighboring wavelength bands are strongly correlated, and remote bands are complementary [89].

HSI systems are used over a range of electromagnetic frequencies, such as the visible (400–700 nm), near-infrared (NIR: 700–1100 nm), and short-wave infrared (SWIR: 1100–2500 nm) [90,91]. Although some spectral regions (especially the red-edge and near-infrared ones) are consistently reported to be highly informative through various studies, there is no single spectral index or wavelength that can be said to be universally optimal. This is because there is variability in plant species, type of pathogen, stage of disease, and environmental conditions [92,93]. These strengths notwithstanding, hyperspectral imaging generates high-dimensional images with an intrinsic redundancy, thus being difficult to analyze. Hence, cutting-edge data analytics, such as machine learning [60,94,95], deep learning [53,96,97], and dimensionality reduction techniques such as principal component analysis (PCA) [98,99], are essential for extracting meaningful information and enabling accurate plant disease detection.

### 4.2. Types and Platforms of Hyperspectral Imaging Sensors

The performance of a hyperspectral sensor and the platform of acquisition, to a large extent, dictate the spatial resolution, scale of measurement, throughput of data, and, thus, performance in the detection of diseased plants [90]. Table 7 outlines conventional hyperspectral platforms and applications. while the electromagnetic frequencies of various devices are illustrated in Table 8. Close-range systems are highly accurate and can be utilized to conduct a fine analysis of agriculture, though the performance of these types of systems is sensitive to environmental factors, such as the variability of illumination and shadowing, which compromise the quality of images that they capture [100,101,102]. Subtle biochemical variations provide early disease stages, whereas later stages entail general spectral reactions. This makes spectral selection context-dependent, and there is no optimum wavelength configuration.

#### 4.2.1. Hyperspectral Sensors

Hyperspectral sensors fall into two main categories: non-imaging and imaging.

**Figure 6:**
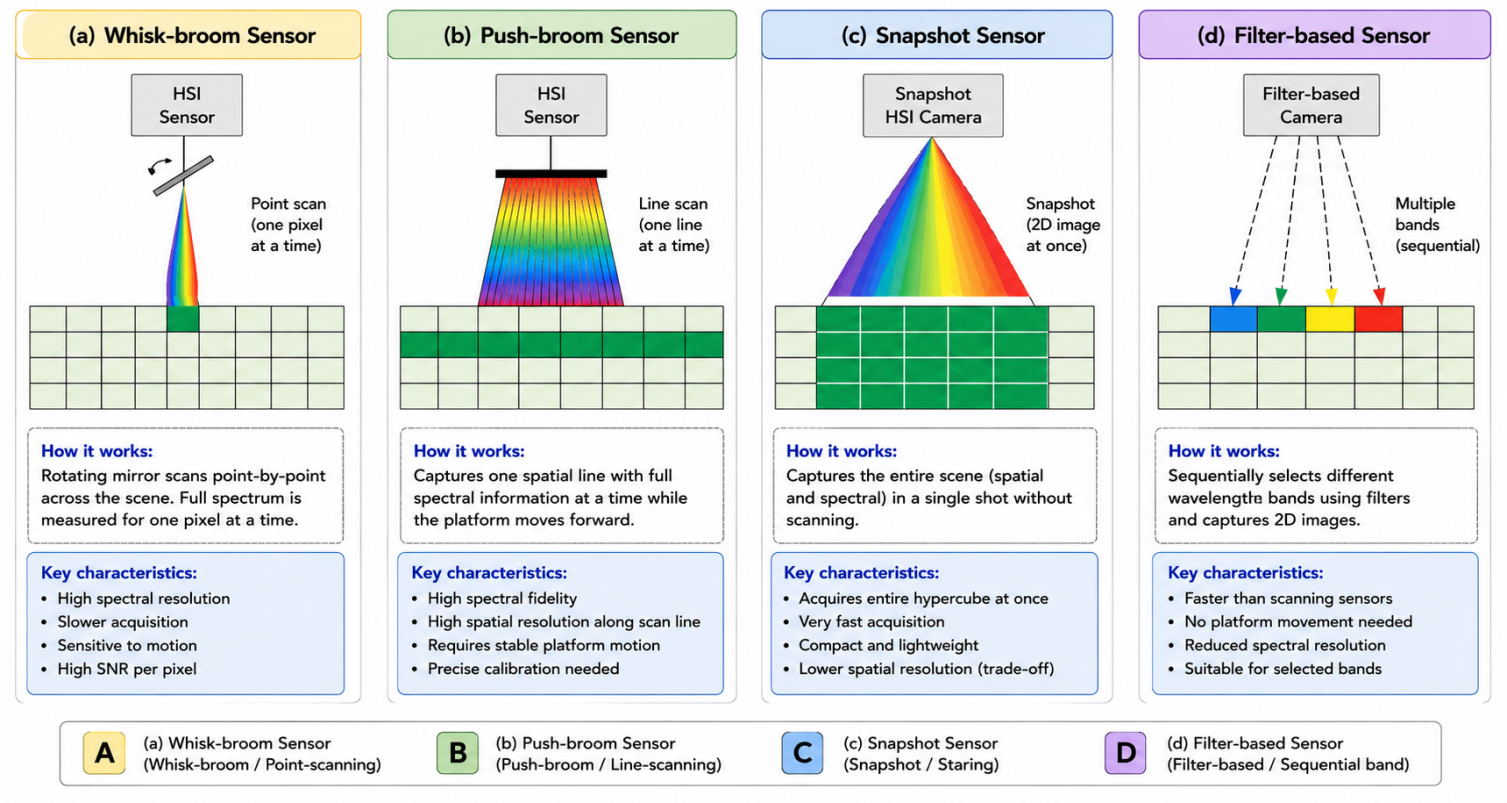
Overview of image acquisition techniques using hyperspectral image (HSI) sensors.

1. Non-imaging sensors:such as spectrometers, measure the average reflectance of a target without capturing spatial details. They are portable, easy to use, and offer high spectral resolution over a broad range (300–2500 nm) [103,104,105]. However, because they lack spatial information, they also struggle to detect localized disease symptoms that appear at small scales [90].
2. Imaging sensors: capture both spatial and spectral information, forming a three-dimensional hypercube where each pixel holds a unique spectral signature [103]. They operate using four main modes, as shown in Figure 6.
  (a) Whisk-broom sensors: scan point-by-point using a rotating mirror, providing high spectral resolution at the cost of slower acquisition [6,90,106,107].
  (b) Push-broom sensors: capture one spatial line with full spectral information at a time, offering high spectral fidelity but requiring precise calibration and stable platform motion [106,107,108,109].
  (c) Snapshot sensors: acquire the entire hypercube at once, resulting in compactness and suitability in dynamic scenes such as UAV applications, but with lower spatial resolution in general [103,110].
  (d) Filter-based sensors: sequentially select image wavelengths, enabling faster acquisition without platform movement, though with reduced spectral resolution [90,107,108].

Push-broom and whisk-broom sensors are good when used on a large scale with aerial and satellite observations, filter-based sensors are used when targeted detection is required, and snapshot sensors are the most versatile in dynamic conditions [103,111].

#### 4.2.2. Hyperspectral Imaging Platforms

**Figure 7:**
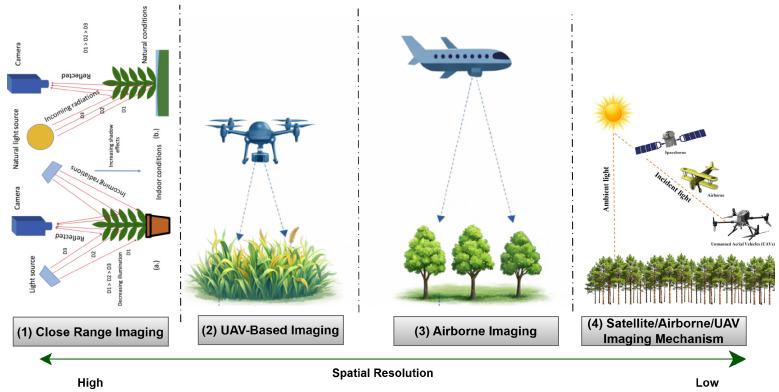
Overview of commonly used hyperspectral imaging platforms for plant disease detection, including (1) close-range (ground-based) where (a) indoor imaging with artificial illumination showing decreasing light intensity with distance (D1 > D2 > D3), and (b) outdoor imaging under natural sunlight with varying shadow and illumination effects, (2) UAV-based, (3) airborne, and (4) satellite-based systems. The illustration was intuitively redrawn based on the concept presented in [112].

Hyperspectral sensors are deployed on various platforms, each offering distinct spatial, spectral, and temporal characteristics [100]. Furthermore, Figure 7 visually summarizes these systems; further, a brief description of each platform is given below.

1. Close-range platforms (ground-based): Give high spatial resolution, allowing a detailed analysis of leaf structure and early disease symptoms. These systems usually employ regulated configurations, but field conditions may bring in variability of illumination [100,101,102,113].
2. UAV-based platforms: Provide cost-effective and flexible high-resolution data acquisition. They can be equipped with lightweight sensors that provide low-altitude imaging but are limited by payload capacity, flight time, and environmental requirements [53,100,114,115,116,117].
3. Airborne platforms: Deliver moderate-to-high spatial resolution on broad scales and have many applications in crop health assessment. Nevertheless, they are restricted by high operational expenses and rigorous flight stability considerations, and their availability is limited by factors such as high costs and specific requirements to be met to achieve flight stability [100,117,118,119,120,121].
4. Satellite-based platforms: Support large-scale agricultural monitoring but with relatively coarse spatial resolution (usually 17–36 m) and temporal resolution, limiting their usefulness in detecting diseases at the early stage of development [29,100,122,123,124,125].

### 4.3. Biological and Spectral Basis of Disease-Induced Reflectance

Spectral imaging in detecting plant diseases is essentially dictated by physiological and biochemical changes that take place in the leaf tissues. Such changes have a direct impact on the properties of light absorption, light reflection, and light scattering in various wavelength bands, as shown in the table of spectral physics demonstrated in Table 8.

Photosynthetic pigments dominate reflectance in the visible spectrum (400–700 nm), including chlorophyll and carotenoids. Chlorophyll degradation caused by disease causes a reduction in light absorption in the red region (680 nm), which leads to higher reflectance and visible symptoms like chlorosis. Likewise, alterations in carotenoid make alterations to reflectance in the blue and green wavelengths, which contribute to premature discoloration. Nevertheless, the red edge (700–750 nm) is very sensitive to small changes in the chlorophyll level, which are commonly known as an early sign of plant stress. The red edge position being shifted to shorter wavelengths is usually linked to an occurrence of disease before the onset of manifestations of disease.

The internal structure of the leaf, especially the spongy mesophyll, prevails in the reflectance in the near-infrared range (750–1400 nm). The development of the disease interferes with cell integrity, decreasing internal scattering and causing an apparent decrease in reflectance. Thus, the area is very important in evaluation of structural damage. Moreover, the shortwave infrared (1300–2500 nm) region is correlated with biochemical constituents, lignin, cellulose, and proteins, as well as water content. Disease progression disrupts cellular integrity, reducing internal scattering and leading to a noticeable decrease in reflectance. Therefore, this region is critical for assessing structural damage. Furthermore, the shortwave infrared region (1300–2500 nm) is associated with water content and biochemical components such as lignin, cellulose, and proteins. Disease-induced dehydration and metabolic changes alter absorption features in this region, providing information on advanced stress conditions and tissue degradation.

## 5. Common Plant Diseases

Plant diseases are primarily caused by fungal, bacterial, and viral pathogens, with more than 50,000 documented disorders affecting plant systems worldwide [126]. Once infected, plants exhibit a wide range of symptoms across leaves, stems, and fruits, varying according to the pathogen type and infection stage [127]. From a diagnostic perspective, disease detection methods are categorized into direct and indirect approaches [128]. Direct techniques rely on serological and molecular tools—such as ELISA, PCR, and fluorescence-based assays—to accurately identify pathogens [128,129]. However, these methods are often resource-intensive and may not be suitable for large-scale field monitoring. In contrast, indirect methods detect disease through physiological and biochemical alterations, including changes in transpiration, temperature, morphology, and emitted metabolites [129]. Imaging and spectroscopic techniques, particularly hyperspectral imaging, fall within this category, as they capture subtle variations in plant spectral responses associated with stress and infection.

**Figure 8:**
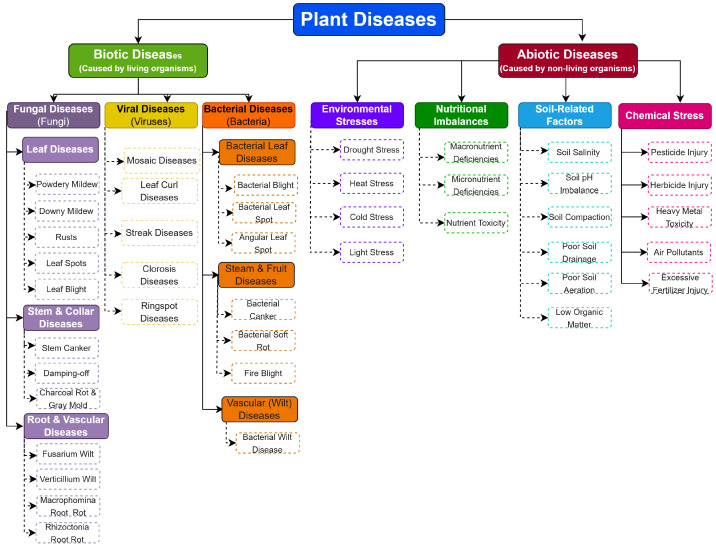
Taxonomic representation of major categories of biotic and abiotic diseases, along with their associated subtypes, that affect plant health and agricultural productivity. Different colors are used to distinguish disease categories and subcategories, improving visual clarity and hierarchical interpretation.

The pathogenesis of diseases usually occurs in three phases: the first step is inoculation (infection), a latent period, during which the pathogenic agents proliferate rapidly, and the last stage is the appearance of visible symptoms. Early symptoms normally manifest themselves in local areas of the foliage and are frequently hard to identify through conventional visual examination [92]. Cereals, tubers, oil seeds, vegetables, and fruits are agronomically highly susceptible to a large range of diseases, with dire economic impacts, as explained in Section 1. However, Figure 8 is a representation of some common taxonomic categories of plant diseases and Table 9 gives a summary of some of the key pairs of crop diseases with significant global impact. The especially important crops to global food security and trade are staple crops like rice, wheat, maize, and barley [19,130], while crops such as potato, soybean, citrus, and others play an essential role in food and industrial supply chains [131,132,133,134,135,136,137,138,139].

Such diseases as rice blast, sheath blight, leaf rust, powdery mildew, fusarium head blight, late blight, and citrus greening may lead to severe loss of production of up to 50 percent, being very dangerous to food security and the global economy in general [140]. These diseases often disrupt plant physiological processes, including chlorophyll content, cellular structure, and water regulation. For instance, fungal infections such as Rhizoctonia solani and *Magnaporthe oryzae* degrade photosynthetic tissues, while pathogens like Puccinia triticina and Botrytis cinerea impair plant metabolism and structural integrity [141,142,143,144,145]. Viral diseases, including potato virus Y and tomato yellow leaf curl virus, introduce complex symptom variability due to strain diversity and vector-based transmission [146,147]. Similarly, bacterial infections such as citrus canker and sour skin lead to tissue degradation and post-harvest losses [148,149,150,151].

**Table 9:** Overview of major plant diseases affecting agricultural crops, categorized by disease type and affected plant parts.

Crop Category	Crop	Disease	Pathogen Type	Affected Plant Parts	References
Cereal	Rice	Sheath blight	Fungal	Leaves	[152,153]
	Wheat	Leaf rust	Fungal	Leaves, stems	[154,155]
		Stripe rust	Fungal	Leaves	[156]
		Powdery mildew	Fungal	Leaves	[157]
		Fusarium head blight	Fungal	Ears, stalks	[158]
	Maize	Common Rust	Fungal	Leaves	[159]
		Northern Leaf Blight	Fungal	Leaves	[159]
		Gray Leaf Spot	Fungal	Leaves	[159]
	Barley	Powdery mildew	Fungal	Leaves	[160]
	Barley	Blumeria graminis f. sp. hordei infection	Fungal	Epidermis, Leaf	[160]
	Sorghum	Anthracnose	Fungal	Leaves, stalks	[161]
	Pearl Millet	Downy mildew	Fungal	Leaves, inflorescence	[161]
Tuber	Potato	Potato virus Y	Viral	Leaves	[162]
		Potato Mosaic Disease	Virus	Leaves	[162]
		Late blight	Oomycete / Fungus	Leaf, Stem, Tuber	[163]
	Sugar beet	Beet rust	Fungal	Leaves	[164]
		Cercospora leaf spot	Fungal	Leaves	[165]
		Powdery mildew	Fungal	Leaves	[166]
		Root rot	Fungal	Leaves, roots	[167]
	Cassava	Cassava mosaic disease	Viral	Leaves	[168]
		Cassava brown streak disease	Viral	Leaves, tubers	[168]
	Sweet Potato	Sweet potato virus disease	Viral	Leaves, tubers	[168]
Oil seed	Canola	Blackleg	Fungal	Leaves, stems, pods	[169]
		Sclerotinia stem rot	Fungal	Stems	[169]
		White leaf spot	Fungal	Leaves	[169]
		Downy mildew	Fungal	Leaves	[169]
	Soybean	Yellow mosaic virus	Viral	Leaves, pods	[170]
		Anthracnose	Fungal	Stems, leaves, pods	[171]
	Sunflower	Sclerotinia stem rot	Fungal	Stems	[172]
		Downy mildew	Fungal	Leaves, stems	[172]
	Sesame	Phyllody	Phytoplasma	Leaves, flowers, pods	[172]
Vegetable	Tomato	Yellow leaf curl	Viral	Leaves	[173]
		Gray mold	Fungal	Leaves, stems, fruits	[174]
	Cucumber	Angular spot	Bacterial	Leaves	[175]
	Capsicum	Verticillium wilt	Fungal	Leaves, stems, roots	[176]
		Phytophthora blight	Fungal	Leaves, fruits, roots	[165]
	Onion	Purple Blotch; Downy Mildew; Stemphylium Leaf Blight; Botrytis Leaf Blight	Oomycete/Fungal	Leaves	[177]
	Eggplant	Verticillium wilt	Fungal	Leaves, stems, roots	[161]
	Carrot	Alternaria leaf blight	Fungal	Leaves, petioles	[161]
Fruit	Citrus	Citrus greening (Huanglongbing)	Bacterial	Leaves, fruits, roots	[178]
		Citrus canker	Bacterial	Leaves, fruits	[178]
	Grapevine	Downy mildew	Fungal	Leaves	[179]
		Powdery mildew	Fungal	Leaves, stems, fruits	[179]
	Apple	Verticillium wilt	Fungal	Leaves, stems, roots	[180]
	Pear	Cercospora leaf spot	Fungal	Leaves	[165]
	Blueberries	Leaf mottle	Viral	Leaves, fruits	[181]
	Plum	Fusarium fruit rot	Fungal	Fruits	[182]

**Figure 9:**
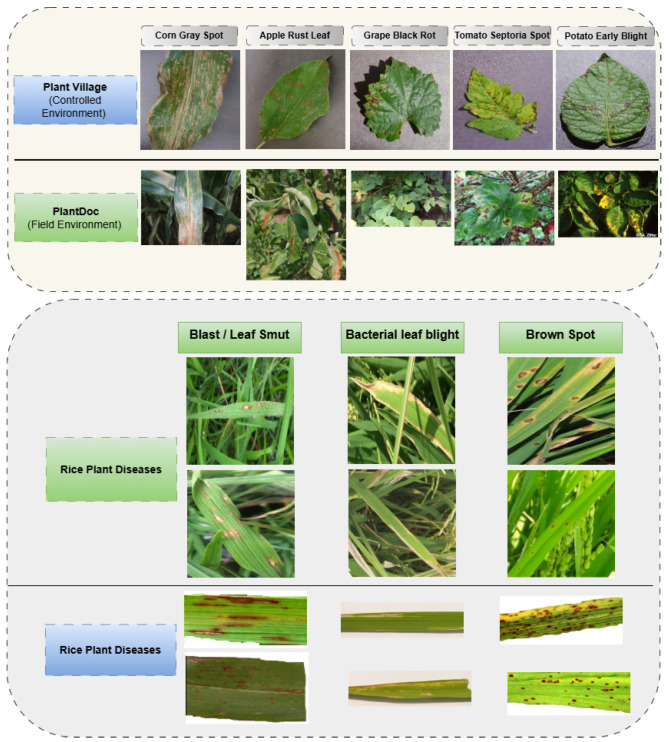
Subsets of common crop disease datasets showing leaf images with visible symptoms from both controlled and field environments.

Figure 9 represents the visual symptoms of the major crop diseases in multiple agricultural categories of common datasets [183,184,185]. The figure also illustrates the complexities and challenges of field-environment datasets. The figure also illustrates disease-specific patterns. Given their widespread prevalence and destructive potential, timely and accurate disease detection is critical. Hyperspectral imaging offers a promising solution by enabling early-stage detection through detailed spectral characterization of plant health. Unlike conventional approaches, it identifies disease-induced physiological changes before visible symptoms appear, making it particularly valuable for precision agriculture applications.

### 5.1. Datasets

The rapid progress of machine learning and deep learning for plant disease detection has been largely driven by the availability of diverse RGB-based benchmark datasets. These datasets span multiple crop species, disease categories, imaging backgrounds, and geographical regions, enabling both controlled laboratory studies and real-world field evaluations. Table 10 provides a consolidated overview of the most widely used publicly available plant disease datasets that support algorithm development, benchmarking, and generalization analysis.

### 5.2. Representative Hyperspectral Imaging Datasets and Experimental Studies

Hyperspectral imaging (HSI) resources are significantly smaller in size and are less accessible to the general public compared to RGB-based plant disease datasets. The majority of HSI research uses self-obtained experimental data collected in laboratory or field settings and UAV-based sensor arrays, and only a handful of spectral libraries and crop-weed standards have been published, as shown in Table 11. Such limited standardization of datasets hinders reproducibility, cross-study comparison, and sound assessment of deep learning models’ performance in real-world agricultural settings. Furthermore, the table extends beyond descriptive reporting by incorporating analytical attributes such as spectral resolution, acquisition environment, and validation strategy. The analysis reveals that most hyperspectral studies are conducted under controlled conditions with limited external validation, which restricts their transferability to real-world agricultural environments.

## 6. Comparative Analysis and Recommendations

### 6.1. Comparative Analysis of Detection Techniques

Table 12, Table 13, Table 14 and Table 15 present a comparative synthesis of plant disease detection approaches across multiple studies. There is substantial heterogeneity in crops, sensing platforms, environmental conditions, and evaluation metrics. The reported values are derived as qualitative or range-based indicators synthesized from multiple sources of the literature.

Specifically, (i) cost ranges are estimated from typical hardware and deployment configurations reported in the literature, (ii) speed reflects approximate processing time categories (e.g., real-time, seconds, minutes), (iii) accuracy is expressed as relative performance levels (e.g., moderate, high) rather than exact percentages, and (iv) scalability and field performance are interpreted based on deployment feasibility reported in prior studies.

As summarized in Table 12, plant disease detection techniques exhibit clear trade-offs across deployment dimensions. RGB-based approaches offer low-cost and highly scalable solutions for routine field applications, whereas hyperspectral frameworks achieve the highest accuracy and strongest early-stage detection capability but remain limited by acquisition cost and slower processing. In contrast, multimodal fusion, edge AI, and smartphone-assisted systems provide a more deployment-friendly balance between robustness, speed, and scalability, highlighting their growing importance for practical precision agriculture.

**Figure 10:**
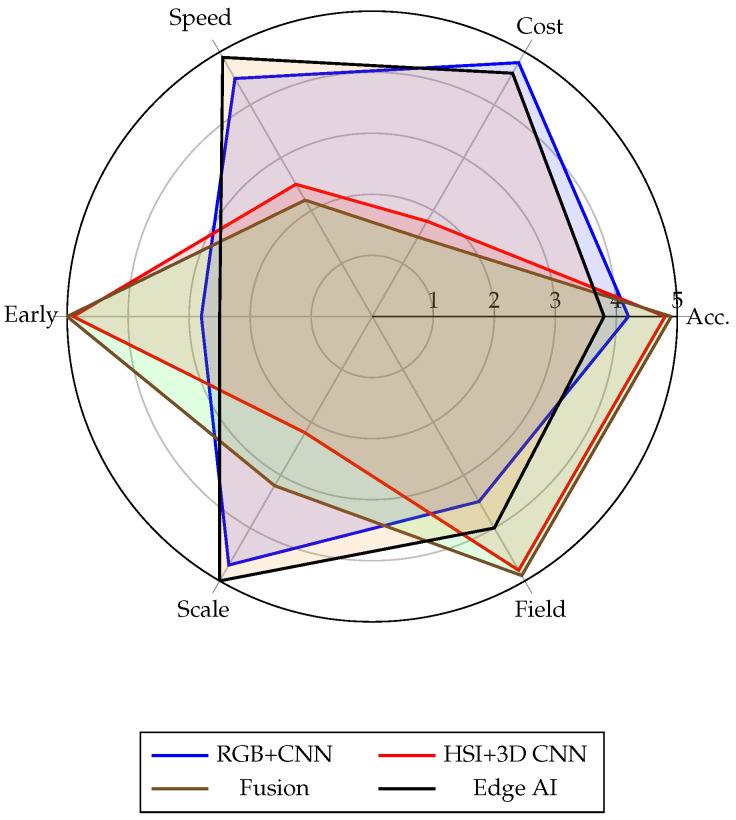
Compact Compact radar chart comparison of major plant disease detection techniques across deployment-oriented criteria, including accuracy (Acc.), computational cost (Cost), processing speed (Speed), early detection capability (Early), scalability (Scale), and field applicability (Field). The colored regions represent different detection approaches: blue (RGB + CNN), red (HSI + 3D CNN), green (Fusion), and orange (Edge AI). Numerical values range from 1 to 5, where higher scores indicate better suitability and performance for the corresponding criterion.

Table 13 and Figure 10 summarize the practical trade-offs between key plant disease detection frameworks based on deployment-oriented dimensions. The growing popularity of RGB-based deep learning models is due to the ability to provide a balance between low cost, fast inference, dataset accessibility, and high scalability, which is suitable for routine field diagnosis and mobile applications. Hyperspectral models, especially HSI + 3D CNN, offer better early and pre-symptomatic detection with high field sensitivity but are expensive to acquire and slow to process, which means they cannot be used on large scales. Radar graph Figure 10 analysis also shows that multimodal RGB-HSI fusion has the best overall performance in terms of accuracy, early detection, and robustness, whereas edge AI gives the best real-time scalability of precision agriculture systems under resource limitations.

### 6.2. Disease Stage vs. Imaging Modality Analysis

Table 14 indicates the appropriateness of the various imaging modalities at different stages of progressive disease. The comparison clearly shows that hyperspectral imaging always exhibits an excellent sensitivity range between pre-symptomatic and severe stages because it is capable of capturing subtle biochemical and physiological changes in the plant images even before observing the visual symptoms. The current study is firmly supported by this stage-level advantage, as it encourages further implementation of HSI-based deep learning models in the context of early and accuracy-focused plant disease detection.

### 6.3. Dataset Benchmark Limitations and Research Gaps

Table 15 shows the limitations of the presently existing RGB and hyperspectral benchmarks datasets. RGB public datasets provide an excellent standardization and effective performance in deep learning models. However, these datasets remain poor in temporal disease evolution and pre-visible disease sensing and detection. Conversely, hyperspectral datasets offer high-quality spectral richness and early disease sensitivity but remain limited by small amounts of publicly available data, limited standardization, and support of multimodal benchmarks, which underscores the pressing need to consolidate large-scale RGB-HSI datasets.

### 6.4. Practical Recommendations for Future Research and Deployment

Based on the comprehensive review conducted in this article, the comparative analyses in Table 13, Table 14 and Table 15, and the deployment-oriented synthesis illustrated in Figure 10, several practical recommendations can be drawn for future research on the detection of plant diseases and deployment in the real world.

R1. RGB-based deep learning frameworks should remain the primary choice for large-scale field deployment, as they provide the best balance between acquisition cost, inference speed, scalability, and the availability of well-established public benchmarks.

R2. For pre-symptomatic disease detection, biochemical stress assessment, and high-value crop monitoring, hyperspectral imaging integrated with spectral–spatial deep learning models (e.g., 3D-CNN and transformer-based spectral networks) is recommended as the most reliable and sensitive solution.

R3. Future intelligent crop monitoring systems should prioritize multimodal fusion of RGB, hyperspectral, thermal, UAV, and environmental sensing modalities to improve robustness to illumination variability, occlusion, disease co-occurrence, and complex field conditions in real-world environments.

R4. A critical limitation is an insufficient number of standardized and publicly available hyperspectral plant disease benchmarks that include temporal progression labels, which currently limits reproducibility, fair benchmarking, and the creation of generalized deep learning models.

R5. For resource-constrained precision agriculture environments, lightweight vision transformers, mobile CNNs, and edge AI frameworks can be optimized for smartphone-assisted, UAV-assisted, and IoT-enabled real-time disease diagnosis.

R6. Future dataset development efforts can emphasize large-scale RGB-HSI paired benchmarks with disease-stage annotations, temporal continuity, and geographically diverse field conditions to accelerate robust multimodal learning and cross-domain generalization.

## 7. Conclusions

Plant disease detection has evolved from conventional RGB-based feature engineering toward deep learning-driven frameworks that increasingly incorporate spectral–spatial intelligence beyond the visible spectrum. This review synthesized this progression through taxonomy development, bibliometric analysis (2008–2026), dataset evaluation, and deployment-oriented comparisons. The analysis confirms that RGB-based deep learning frameworks remain the most practical solution for large-scale agricultural deployment, offering a favorable balance between cost, inference speed, scalability, and the availability of well-established public benchmarks. Conversely, hyperspectral imaging plays a vital role in early disease diagnosis and pre-symptomatic detection of diseases as it can record biochemical and physiological alterations that cannot be seen in RGB pictures.

In spite of these developments, there is still a big discrepancy between laboratory performance and practical implementation. Optimized models performed under controlled conditions tend to be less robust in field conditions, such as in response to illumination, environments with complexity, and geographic variations. Moreover, hyperspectral imaging systems have issues associated with the high cost of acquisition, high dimensionality of data, and computational requirements. The absence of standardized, publicly available hyperspectral tests, especially with an annotation of disease progression over time, remains an impediment to reproducibility, equitable comparison, and generalized model creation.

In order to overcome these constraints, future studies need to focus on deployment-oriented and system-level innovation. To begin with, deep learning based on RGB has to remain the main platform on which scalable field applications are based, and hyperspectral imaging has to be used selectively on high-value crops, biochemical stress analysis, and early disease detection where a higher degree of sensitivity is needed. Second, multimodal sensing frameworks incorporating RGB, hyperspectral, thermal, UAV-based, and environmental measurements need to be developed to be more resilient to real-world issues like occlusion, variability in illumination, and presence of co-occurring diseases. Third, lightweight and edge-deployable models, such as mobile CNNs, vision transformers, and edge AI systems, need to be optimized to real-time applications on smartphones, UAVs, and IoT-enabled platforms.

Moreover, the development of standardized datasets should be made a research priority. Publicly available, large-scale, RGB-HSI paired datasets with temporal annotations, disease-stage labeling, and geographically varied field conditions are essential to support fair benchmarking, reproducibility, and cross-domain generalization. In addition to this, it is important to consider the use of domain adaptation and generalization strategies, such as self-supervised learning and foundation models, to increase the robustness of models in different environmental settings. Lastly, plant pathologists, agronomists, and AI researchers must work together as an interdisciplinary team to make sure that the development of computational capabilities is matched by biological and practical agricultural requirements.

Multimodal sensing, scalable AI architecture, and deployment-ready system design are the future of plant disease detection. By continuing down these well-established research paths, it will be possible to create effective, interpretable, and real-time diagnostic systems that can sustainably produce worldwide food production and allow precision agriculture.

## Figures and Tables

**Table 2:** Comparison of related survey studies across sensing modalities, gap awareness, multimodality, and LLM/foundation model coverage [✔ shows contribution availability against particular column, while ✘ represents unavailability or unspecified contribution against mentioned column category].

Study Ref. & (YoP)	RGB	HSI	Lab—Field Gap	Multimodal	LLMs
*First Generation: Task-Specific Reviews (pre-2022)*					
[15] & (2016)	✔	✘	✘	✘	✘
[50] & (2018)	✔	✘	✘	✘	✘
[51] & (2018)	✔	✔	✘	✘	✘
[52] & (2020)	✔	✔	✘	✘	✘
*Second Generation: Multimodal & Gap-Aware Reviews (2022–2024)*					
[53] & (2022)	✔	✔	✔	✘	✘
[18] & (2024)	✔	✘	✔	✘	✘
[54] & (2024)	✘	✔	✔	✔	✘
[55] & (2024)	✔	✔	✔	✘	✘
*Third Generation: LLM & Generative AI Reviews (2025–present)*					
[49] & (2025)	✔	✔	✘	✔	✔
[22] & (2025)	✔	✔	✔	✔	✘
[56] & (2025)	✔	✔	✔	✘	✘
[57] & (2025)	✔	✔	✔	✔	✘
[58] & (2025)	✔	✔	✔	✔	✘
This Work	✔	✔	✔	✔	✔

**Table 3:** Temporal evolution of scientific contributions in plant disease diagnosis from 2008 to 2026. The 2026 value represents an annualized projection derived from the first-quarter publication count using Equation (2).

Year	Publications	Growth (%)
2008	20	–
2009	30	50.0
2010	45	50.0
2011	55	22.2
2012	65	18.2
2013	80	23.1
2014	95	18.8
2015	110	15.8
2016	130	18.2
2017	160	23.1
2018	191	19.4
2019	320	67.5
2020	532	66.3
2021	882	65.8
2022	1780	101.8
2023	1960	10.1
2024	3310	68.9
2025	5455	64.8
2026	6120	12.2

**Table 4:** Country-wise research contribution in the field of plant disease detection.

Country	(%)
China	28
USA	22
India	15
Germany	10
UK	8
Others	17

**Table 5:** Lab-to-Field Performance Gap in Conventional RGB-Based Plant Disease Detection Using DL.

Model	PlantVillage Acc. (%)	PlantDoc/Field Acc. (%)	Acc Drop (%)	Reference
VGG16	92.85	42.32	50.53	[6]
InceptionResNetV2	92.96	58.59	34.37	[6]
InceptionV3	71.85/99.22	44.82/65.0	27.03/34.22	[6,77]
Inception Conv ViT	99.94	77.54	22.40	[78]
ConvNeXt	99.47	71.76	27.71	[22]
ViT	99.95	69.0	30.95	[77]
Ensemble	99.96/99.69	68.0/60.0	31.96/39.69	[77,79]
EfficientNet	99.22	59.0	40.22	[77]
MobileNet	99.24	73.86	25.38	[6]
CNN	99.53	33.27	66.26	[50]
CNN	99.84	39.38	60.46	[80]
CNN	99.01	45.95	53.06	[81]
ResNet50	98.18	41.79	56.39	[22]
Dual-stream HBP	98.91	75.06	23.85	[82]
Tri-Linear CNN	99.99	85.80	14.19	[83]
API-Net	99.93	69.0	30.93	[83]
TransFG	99.95	68.0	31.95	[77]

**Table 6:** Effects of lab-to-field performance gap in RGB-based plant disease detection.

Factor/Cause	Observed Effect on Models	Quantified Impact (from Table 5)	Insight
**Domain Shift (Lab to Field)**	Significant drop in accuracy across all architectures	Accuracy drop ranges from 14.19% to 66.26%	Core reason for performance degradation: models fail to generalize
**Controlled vs. Real Environment**	Models trained on clean backgrounds fail in complex scenes	CNN models show extreme drops (53–66%)	Simpler architectures are more sensitive to environmental noise
**Illumination Variability**	Feature inconsistency and misclassification	Performance drops ∼25–50% in most models (e.g., VGG16: 50.53%)	RGB models heavily depend on lighting conditions
**Background Clutter & Occlusion**	Reduced precision and increased false positives	Field accuracy often falls below 60% (e.g., ResNet50: 41.79%)	Lack of robustness to real-world agricultural scenes
**Model Overfitting to Lab Data**	High lab accuracy but poor field transferability	Many models achieve >99% lab accuracy but <70% in field	Indicates overfitting and lack of generalization
**Architecture Sensitivity**	Performance varies significantly across models	Advanced models (Tri-Linear CNN: 14.19% drop) outperform basic CNNs (>60% drop)	Model design influences robustness but does not eliminate the gap

**Table 7:** Configuration of some common hyperspectral imaging platforms [100].

Hyperspectral Platforms	Operating Spectral Range (nm)	Spatial Resolution (m)	Spectral Resolution (nm)	Temporal Resolution (days)	Operating Distance (km)	Manufacturer & Country
**Close-range Imaging Platforms**						
American Society for Photogrammetry and Remote Sensing (ASPRS)	-	0.0001–0.01	-	Depends upon operation/flight time	<10 m	-
All Seeing Eye OCT^TM^	600–1000	0.10–0.5	3–15	Depends upon operation/flight time	-	BaySpec, Inc., USA
SNAPSCAN	1100–1700	0.16–0.3	100 bands	Depends upon operation/flight time	-	imec, USA & Netherlands
**UAV-based Imaging Platforms**						
Headwall	400–1000 (VNIR)	0.01–0.5	6 nm–2.5 μm	Depends upon operation/flight time	<0.15	Headwalls, Photonics, USA
UHD-185 Firefly	450–950	0.01–0.5	4	Depends upon operation/flight time	<0.15	Cubert, Germany
**Airplane-based Imaging Platforms**						
CASI	380–1500	1–20	<3.5	Depends upon operation/flight time	1–20	Itres, Canada
AVIRIS	400–2500	36	17	-	-	Jet, PL, USA
Airborne Imaging Spectrometer for Applications (AISA)	400–970 (Eagle)	1–20	3.3	Depends upon operation/flight time	1–20	Specim, Finland
Hyperspectral Mapper (HyMap)	440–2500	-	15	Depends upon operation/flight time	1–20	IS, Australia
**Satellite-based Imaging Platforms**						
Hyperion	357–2576	30	10	16–30	7.7	NASA, USA
PROBA-CHRIS	415–1050	17	34	8	14	ESA, UK
HJ-1 A	0.43–0.90 μm	100	5	2	360	AoST, China
PRISMA	420–2450	30	12	<14	614	ASI, Italy
ENMAP	420–2450	30	6.5	27	30	OHB, System, AG Germany
HyspIRI	380–2500	30–60	10	16	-	NASA, USA

**Table 8:** Relationship between spectral wavelengths, physiological changes, and disease symptoms in plants.

Wavelength Region (nm)	Physiological Change	Spectral Effect	Disease Symptom	HSI Advantage	Recommended DL Method
Visible (400–500) (Blue)	Variation in carotenoids and accessory pigments	Changes in absorption; slight increase in reflectance with pigment degradation	Early stress signals, discoloration	High sensitivity to pigment degradation	1D-CNN
Visible (500–600) (Green)	Reduction in chlorophyll concentration	Increased reflectance (green peak becomes more pronounced)	Chlorosis (yellowing of leaves)	Qualifies chlorophyll loss before visible yellowing	ID-CNN
Visible (600–700) (Red)	Chlorophyll degradation due to infection	Reduced absorption, leading to increased reflectance near 680 nm	Severe chlorosis and necrotic onset	Measure absolute chlorophyll content via reflectance dip	2D-CNN (spatial features)
Red edge (700–750)	Shift in chlorophyll content and photosynthetic activity	Red edge position shifts toward shorter wavelengths (“blue shift”)	Early disease detection before visible symptoms	Most sensitive region for pre-symptomatic stress	3D-CNN
Near-Infrared (750–1300)	Disruption of internal leaf cellular structure (spongy mesophyll)	Reduced scattering → decreased reflectance	Tissue damage, necrosis progression	Reveals structural damage invisible to RGB	ViT or ResNet-3D
Shortwave Infrared (1300–1800)	Changes in leaf water content and biochemical compounds	Altered absorption features related to water bands	Dehydration, wilting	Quantifies water stress via specific absorption bands	1D-CNN + LSTM (temporal)
Shortwave Infrared (1800–2500)	Variation in lignin, cellulose, and protein content	Changes in absorption signatures due to biochemical composition	Advanced disease stages, structural degradation	Identifies advanced biochemical breakdown	Multimodal Fusion (HSI + Thermal)

**Table 10:** Publicly available plant leaf disease datasets with geographic coverage and critical analysis (Cls = classes; Bg = background; Label = label quality; Bias = sampling bias; Gen. = generalization).

Dataset	Plants	Cls	Images	Bg	Region	Label	Bias	Gen.	Limitations	Ref.
PlantVillage	Multi-crop	38	54,309	Lab	USA/CH	High	Lab	Low	No field variability	[15]
PlantDoc	Multi-crop	30	2569	Both	USA/CH	Medium	Mixed	Medium	Limited size	[15]
Apple Dataset	Apple	4	1821	Field	USA	Medium	Field	Medium	Small dataset	[71]
PDDB	18 species	171	2326	Both	USA	Medium	Mixed	Medium	Class imbalance	[71]
XDB	18 species	105	46,313	Lab	USA	High	Lab	Low	Controlled conditions	[186]
PlantVillage Ext.	25 species	58	87,848	Both	Global	High	Mixed	Medium	Domain inconsistency	[187]
Citrus Dataset	Citrus	5	759	Field	USA	Medium	Field	Medium	Limited samples	[188]
PDD271	Fruits	271	220,592	Field	China	Medium	Field	High	Label noise	[189]
PlantCLEF 2022	Multi	80	4M	Field/Web	Global	Low	Mixed	High	Noisy labels	[190]
Cassava Dataset	Cassava	5	23,958	Field	Uganda	High	Field	High	Crop-specific	[191]
Tomato-Village	Tomato	9	18,162	Lab	India	High	Lab	Low	No field data	[192]
NGLD Dataset *	Grape	4	27,26	Field with uniform Bg.	India	Medium	RGB Images	Medium	Single-region, limited geographic diversity	[193]
Mango Dataset	Mango	7	3600	Field	Bangladesh	Medium	Field	Medium	Small dataset	[194]

* NGLD (Niphad Grape Leaf Disease Dataset).

**Table 11:** Representative hyperspectral imaging datasets and experimental studies used for plant disease detection and crop monitoring with analytical attributes. (Abbreviations: Env. = environment; Spec. = spectral; Val. = validation; Ref. = Reference).

Dataset	Crop	Bands	Range	Platform	Type	Env.	Spec.	Val.	Limitations	Ref.
Apple Scab HSI	Apple	150+	400–1000	Prox.	Exp.	Lab	Dense	Internal	Controlled conditions	[195]
Soybean Rot HSI	Soybean	240	383–1032	Prox.	Exp.	Lab	Dense	Internal	Lab bias	[171]
Rice Disease HSI	Rice	150+	400–1000	Field	Exp.	Field	Moderate	Limited	Env. variability	[196]
UAV Potato HSI	Potato	100+	400–1000	UAV	Exp.	Field	Moderate	Limited	Low spatial res.	[197]
Field Crop	Multi	150+	350–2500	Proximal Sensors	Review	Field	High	Spectral	Calibration & environmental sensitivity	[198]
LOPEX93 Library	Leaf	200+	400–2500	Spectro.	Library	Lab	Dense	None	No disease labels	[199]
PROSPECT Leaf Spectral Database	Leaf	200+	400–2500	Spectrometer	Spectral Library	Lab	Dense	Pigment-Based	Limited field realism & environmental variability	[200]

**Table 12:** Comparative evaluation of plant disease detection techniques across deployment factors. Values represent approximate ranges and qualitative trends synthesized from multiple studies and should be interpreted as comparative indicators rather than exact benchmarks.

Technique	Cost	Speed	Accuracy	Scalability	Ref.
Classical RGB (Color, GLCM)	Low	Real-time	Moderate	High	[201]
RGB + DL (CNN, ResNet, ViT)	Moderate	Sub-second	Moderate–High	Moderate	[15]
RGB + Vision–Language Models	Moderate–High	Seconds	Moderate	Low	[22]
Hyperspectral Approaches					
HSI + Classical (Indices, PCA)	High	Seconds–Minutes	High	Low	[202]
HSI + Spectral ML	High	Moderate	High	Limited Field Scalability	[203]
HSI + DL (3D-CNN, Spectral Nets)	Very High	Minutes	Very High (reported)	Very Low	[171]
Multimodal Approaches					
Multispectral Imaging-Based Detection	Moderate	Moderate	Moderate	Moderate	[204]
IoT + Environmental Sensors	Moderate	Real-Time	Moderate	Scalable	[205]
UAV Systems (RGB/Multispectral)	Moderate–High	Field-dependent	Moderate	High	[206,207]
Edge and Mobile Systems					
Smartphone Applications	Low	Seconds	Moderate	Very High	[208,209]
Edge AI (Jetson, Coral)	Low–Moderate	Sub-second	Moderate–High	High	[210]
IoT Sensor Networks	Low (per node)	Continuous	Moderate	Very High	[211]

**Table 13:** Performance vs. deployment trade-off analysis of plant disease detection techniques. All values represent qualitative trends synthesized from multiple studies and should be interpreted as comparative indicators rather than exact quantitative measures. (Abbreviations: Det. = detection; Scalab. = scalability; Exp. = explainability).

Technique	Modality	Early Det.	Accuracy	Cost	Speed	Field Suit.	Exp.	Scalab.	Best Use
RGB + CNN	RGB	Moderate	Moderate–High	Low	Fast	Moderate	Moderate	High	Field diagnosis
RGB + ViT	RGB	Moderate	Moderate–High	Moderate	Moderate	Moderate–High	Low	Moderate	Complex symptoms
HSI + SVM	HSI	High	High	High	Moderate	High	High	Low	Early stress detection
HSI + 3D CNN	HSI	Very High	Very High (reported)	Very High	Slow	Very High	Moderate	Low	Pre-symptomatic detection
RGB + HSI Fusion	Fusion	Very High	Very High (reported)	Very High	Slow	Very High	Moderate	Moderate	High-confidence diagnosis
UAV-HSI	HSI	High	High	Very High	Moderate	High	Low	High	Field-scale monitoring
Edge AI	RGB/MSI	Moderate	Moderate	Low	Very Fast	Moderate–High	Moderate	Very High	Edge deployment
Smartphone Apps	RGB	Low	Moderate	Very Low	Very Fast	Moderate	High	Very High	Farmer-level diagnosis

**Table 14:** Comparative suitability of imaging modalities across different plant disease progression stages. (Abbreviations: RGB = red, green, blue; MSI = multispectral; HSI = hyperspectral imaging).

Disease Stage	RGB	MSI	HSI	Thermal	Best AI Framework
Pre-symptomatic	Poor	Moderate	Excellent	Good	HSI + DL
Early visible	Good	Good	Excellent	Good	RGB + CNN
Mid-stage progression	Good	Good	Excellent	Very Good	RGB + HSI Fusion
Severe stage	Excellent	Good	Excellent	Moderate	RGB + ViT
Late-stage tissue damage	Excellent	Moderate	Very Good	Good	Multimodal Fusion

**Table 15:** A Summary of Critical comparison between public RGB datasets and hyperspectral imaging datasets in terms of benchmark readiness, field realism, and future deep learning suitability. (Abbreviations: Avail. = Availability, Std. = Standardization and DL = Deep Learning).

Dataset Type	Avail.	Std.	Field	Diversity	Temporal	DL	HSI
RGB Public Benchmarks	High	Excellent	Moderate	Very High	Poor	Excellent	Poor
RGB Field Datasets	High	Good	High	High	Poor	Excellent	Poor
RGB Web-scale Datasets	High	Moderate	Moderate	Very High	Poor	Excellent	Poor
HSI Public Libraries	Low	Good	Low	Moderate	Poor	Moderate	Excellent
HSI Experimental Studies	Very Low	Low	Very High	Moderate	Poor	Good	Excellent
UAV-HSI Field Collections	Very Low	Low	Excellent	Moderate	Poor	Good	Excellent
Multimodal RGB–HSI Sets	Low	Moderate	High	High	Very Poor	Very Good	Excellent

## Data Availability

The data supporting the findings of this study are available upon reasonable request from the first author.

## Abbreviations

The following abbreviations are used in this manuscript:

1D-CNN	One-Dimensional Convolutional Neural Network
2D-CNN	Two-Dimensional Convolutional Neural Network
3D-CNN	Three-Dimensional Convolutional Neural Network
AI	Artificial Intelligence
CNN	Convolutional Neural Network
DL	Deep Learning
GLCM	Gray-Level Co-occurrence Matrix
HSI	Hyperspectral Imaging
IoT	Internet of Things
KNN	K-Nearest Neighbor
ML	Machine Learning
MSI	Multispectral Imaging
PCA	Principal Component Analysis
PLS	Partial Least Squares
RGB	Red–Green–Blue Imaging
RF	Random Forest
UAV	Unmanned Aerial Vehicle
ViT	Vision Transformer
VLM	Vision–Language Model
XAI	Explainable Artificial Intelligence
